# Supplementary material for: Bacterial Colonization of Microplastics at the Beaches of an Oceanic Island, Tenerife, Canary Islands
Source: Int J Environ Res Public Health. 2023 Feb 23;20(5):3951. doi: 10.3390/ijerph20053951 (PMC10001659; doi:10.3390/ijerph20053951)
Supplement: Supplementary file 1 [file ijerph-20-03951-s001.zip › ijerph-2203127-supplementary.pdf]

Supplementary Material

Bacterial Colonization of Microplastics at Beaches of an Oceanic Island, Tenerife, Canary Islands

Cintia Hernández-Sánchez [1,2,*], Ángel Antonio Pestana-Ríos [1], Cristina Villanova-Solano [2,3], Cristopher Domínguez-Hernández [2,3], Francisco Javier Díaz-Peña [4], Cristobalina Rodríguez-Álvarez [1], María Lecuona [5] and Ángeles Arias [1]

[1] Department of Preventive Medicine and Public Health, Toxicology, Legal and Forensic Medicine and Parasitology, Health Science Faculty, University of La Laguna (ULL), Campus de Ofra s/n, 38071 Santa Cruz de Tenerife, Spain
[2] Institute of Tropical Diseases and Public Health of the Canary Islands, University of La Laguna (ULL), Avda, Astrofísico Fco. Sánchez, s/n°, 38206 San Cristóbal de La Laguna, Spain
[3] Departmental Unit of Analytical Chemistry, Chemistry Department, Science Faculty, University of La Laguna (ULL), Avda Astrofísico Fco. Sánchez, s/n°, 38206 San Cristóbal de La Laguna, Spain
[4] Department of Animal Biology, Soil Science and Geology, Science Faculty, University of La Laguna (ULL), Avda, Astrofísico Fco. Sánchez, s/n°, 38206 San Cristóbal de La Laguna, Spain
[5] Microbiology and Infection Control Service of the University Hospital of the Canary Islands (HUC), 38071 Tenerife, Spain
* Correspondence: chernans@ull.edu.es

Supplementary Material File S1: Plastic composition of microplastics in different beaches and infrared spectrum.

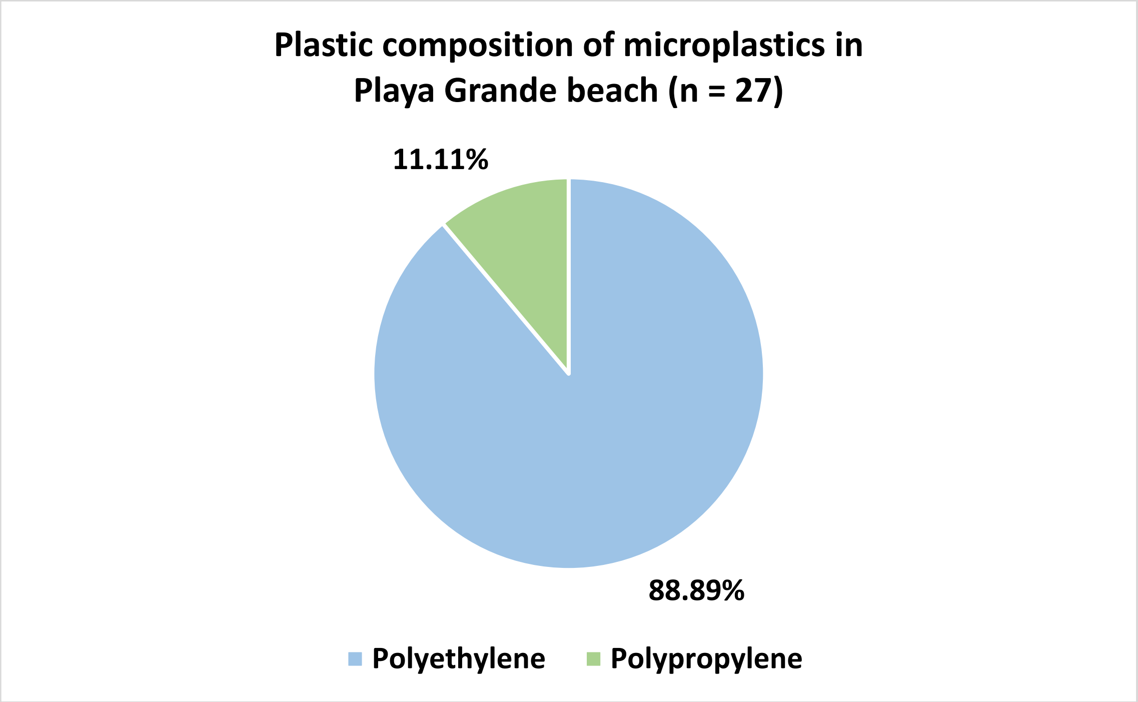
Plastic composition of microplastics in Playa Grande beach (n = 27)
11.11%
88.89%
Polyethylene
Polypropylene

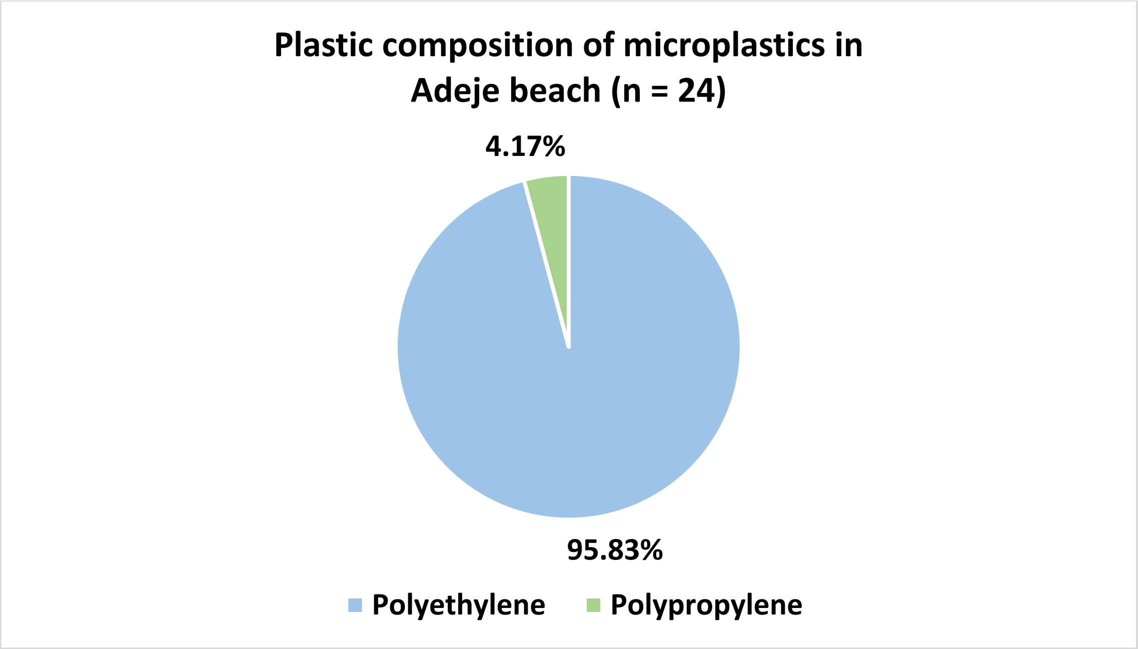
Plastic composition of microplastics in Adeje beach (n = 24)
4.17%
95.83%
Polyethylene
Polypropylene

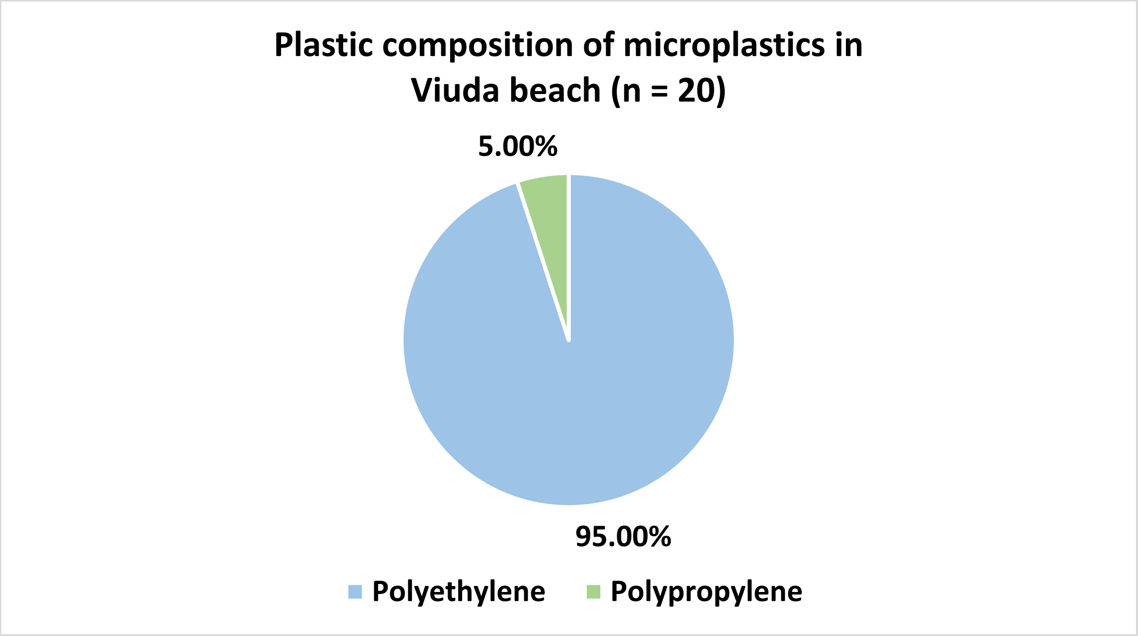
Plastic composition of microplastics in
Viuda beach (n = 20)
5.00%
95.00%
Polyethylene
Polypropylene

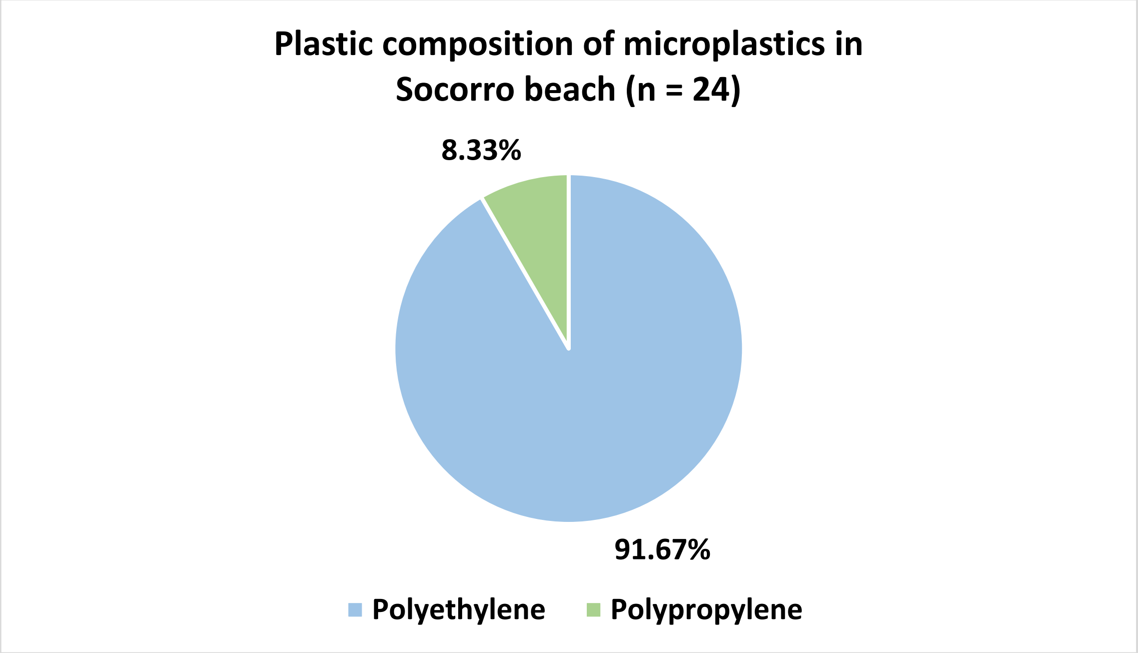
Plastic composition of microplastics in
Socorro beach (n = 24)
8.33%
91.67%
Polyethylene
Polypropylene

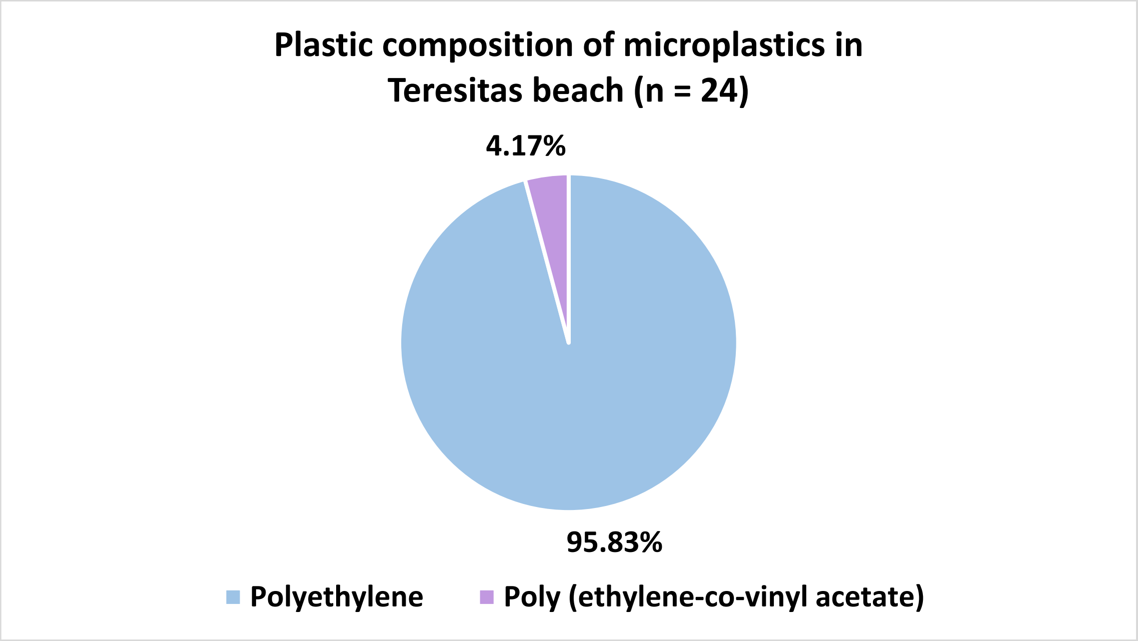
Plastic composition of microplastics in Teresitas beach (n = 24)
4.17%
95.83%
Polyethylene
Poly (ethylene-co-vinyl acetate)

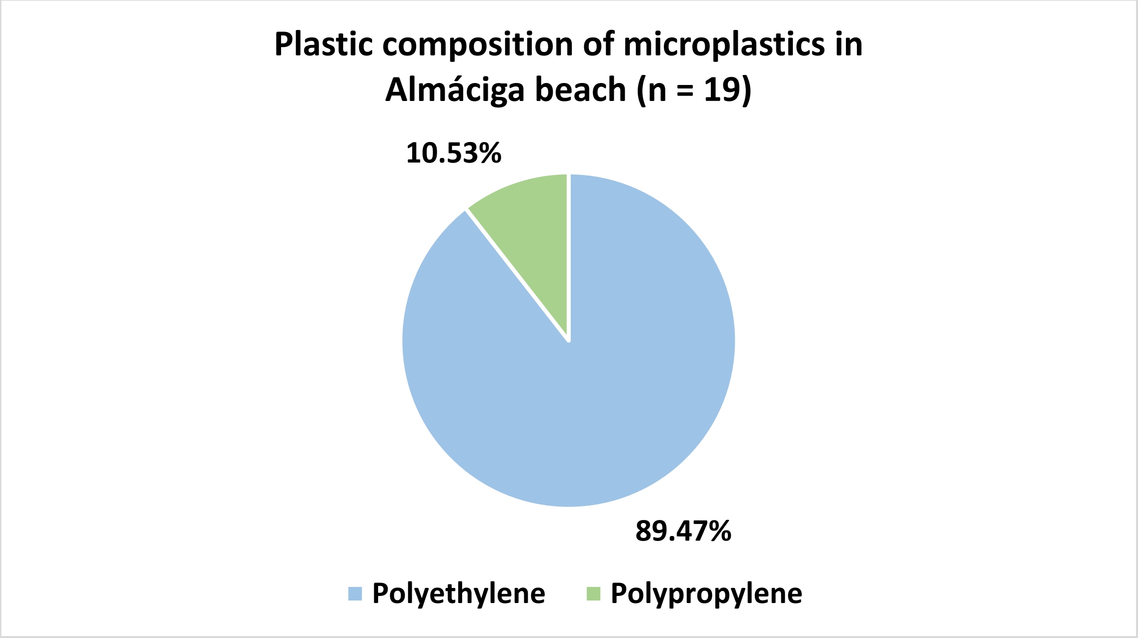
Plastic composition of microplastics in Almáciga beach (n = 19)
10.53%
89.47%
Polyethylene
Polypropylene

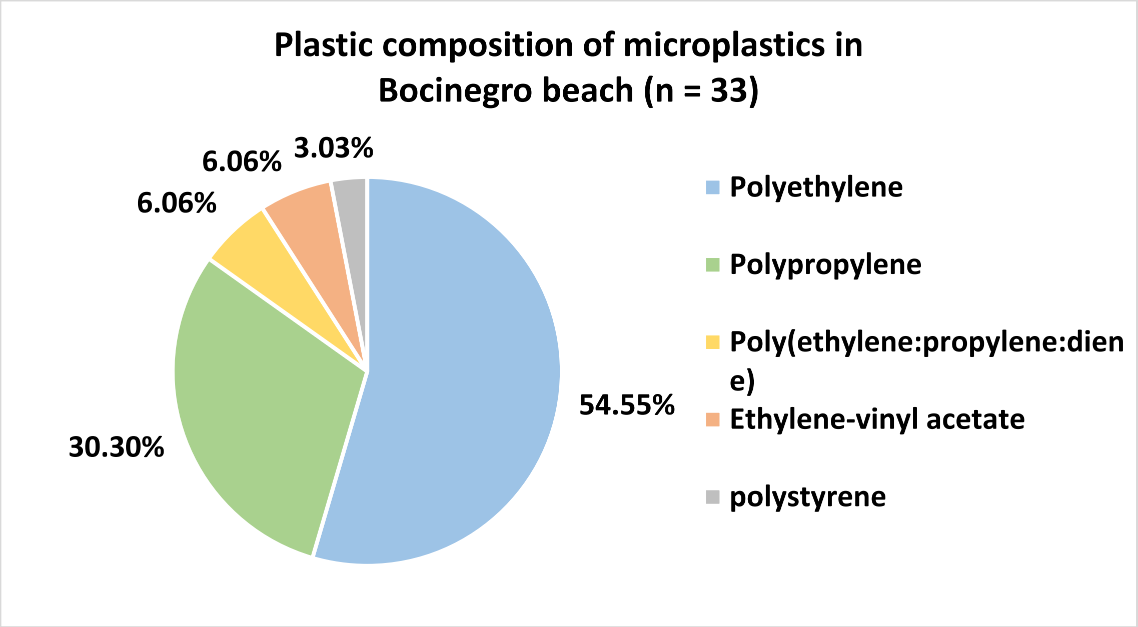
Plastic composition of microplastics in Bocinegro beach (n = 33)
3.03%
6.06%
6.06%
54.55%
30.30%
Polyethylene
Polypropylene
Poly(ethylene:propylene:diene)
Ethylene-vinyl acetate
polystyrene

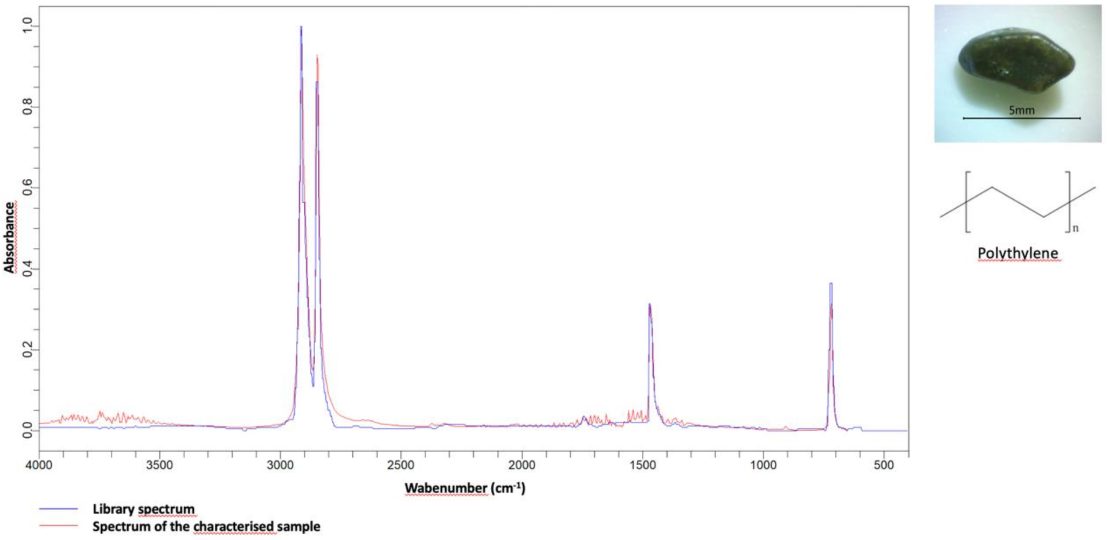

Polyethylene fragment infrared spectrum (matching 96,95 %).

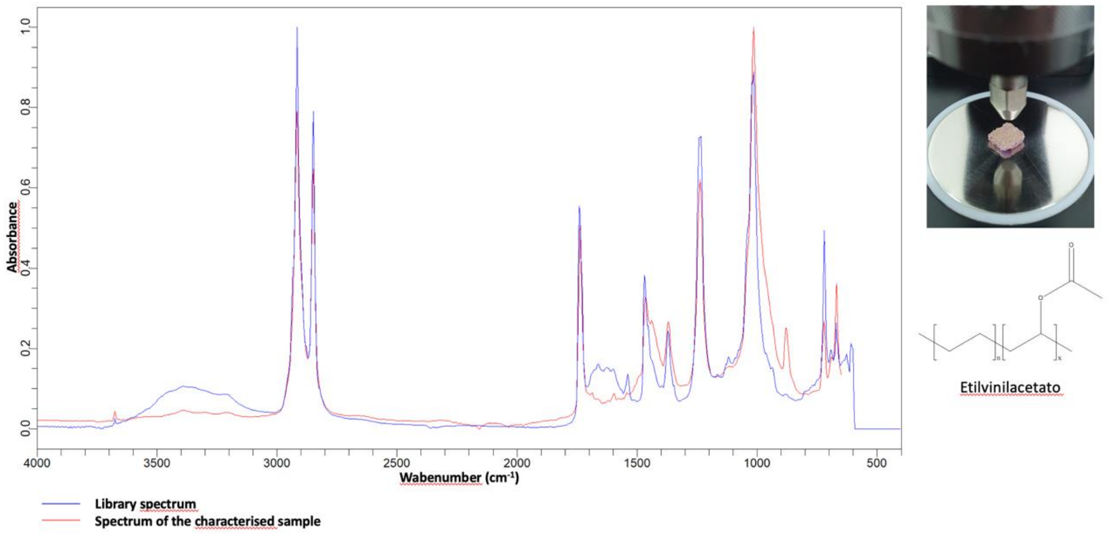

Etilvinilacetato foam infrared spectrum (matching 87,07 %).

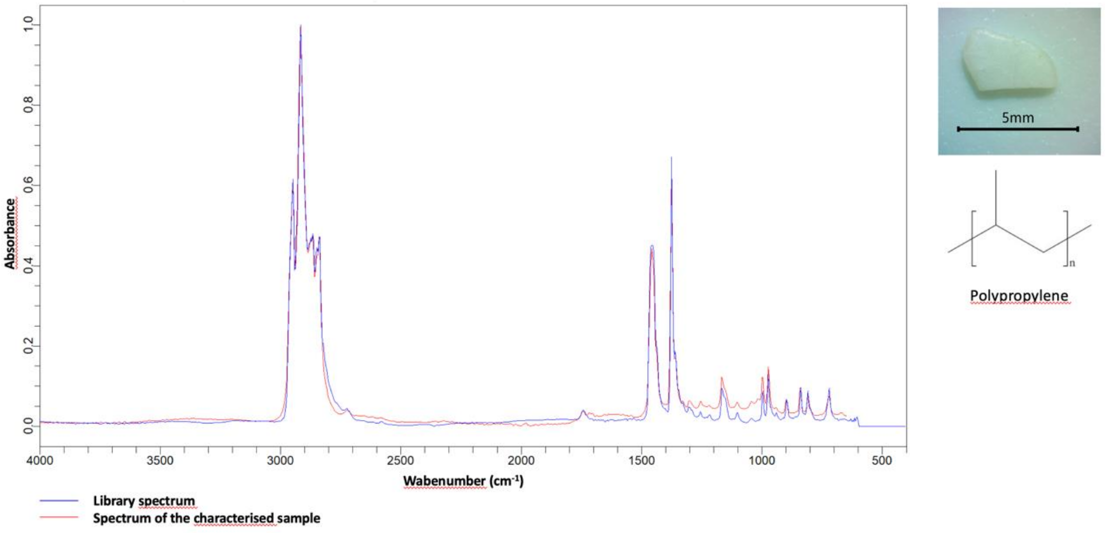

Polypropylene fragment infrared spectrum (matching 98,71 %).

Supplementary Material File S2: Rose of significant wave height of the studied beaches, based on data from Puertos del Estado.

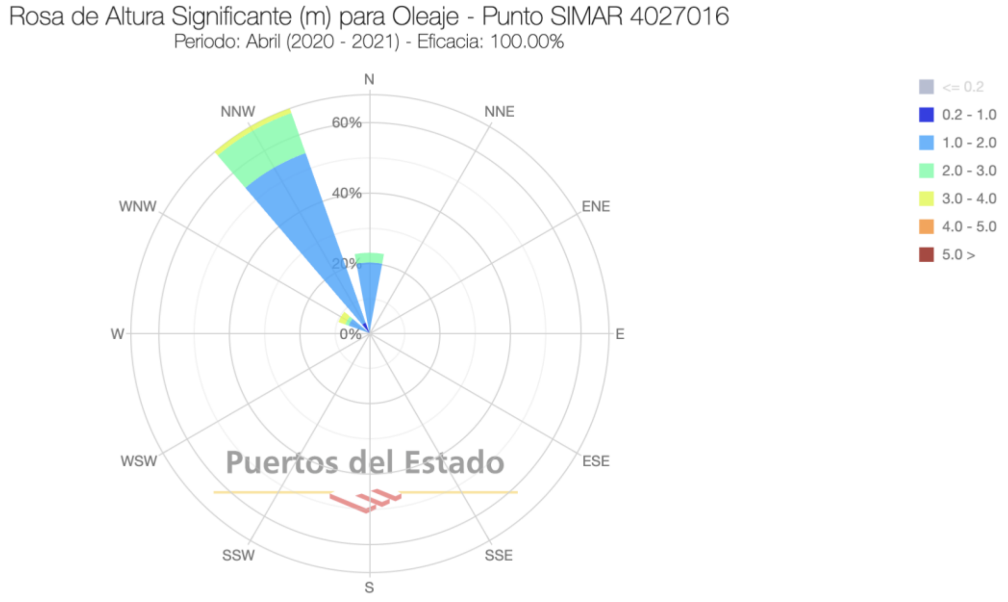

Figure S1: Rose of significant wave height regarding Almáciga beach form April 2020 since April 2021.

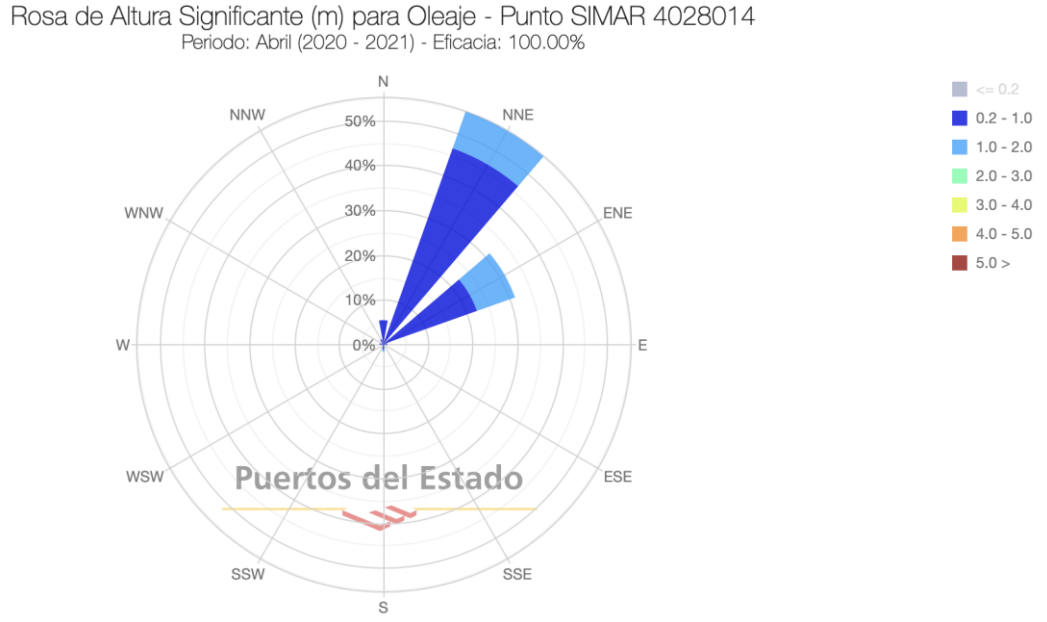

Figure S2: Rose of significant wave height regarding Teresitas beach form April 2020 since April 2021.

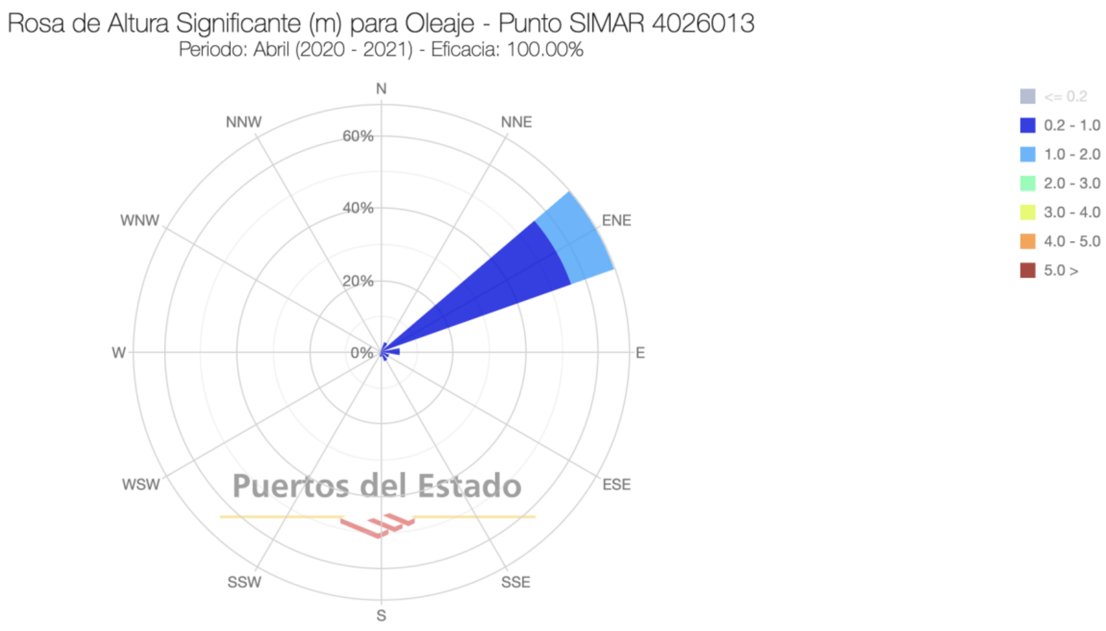

Figure S3: Rose of significant wave height regarding Socorro and Viuda beaches form April 2020 since April 2021.

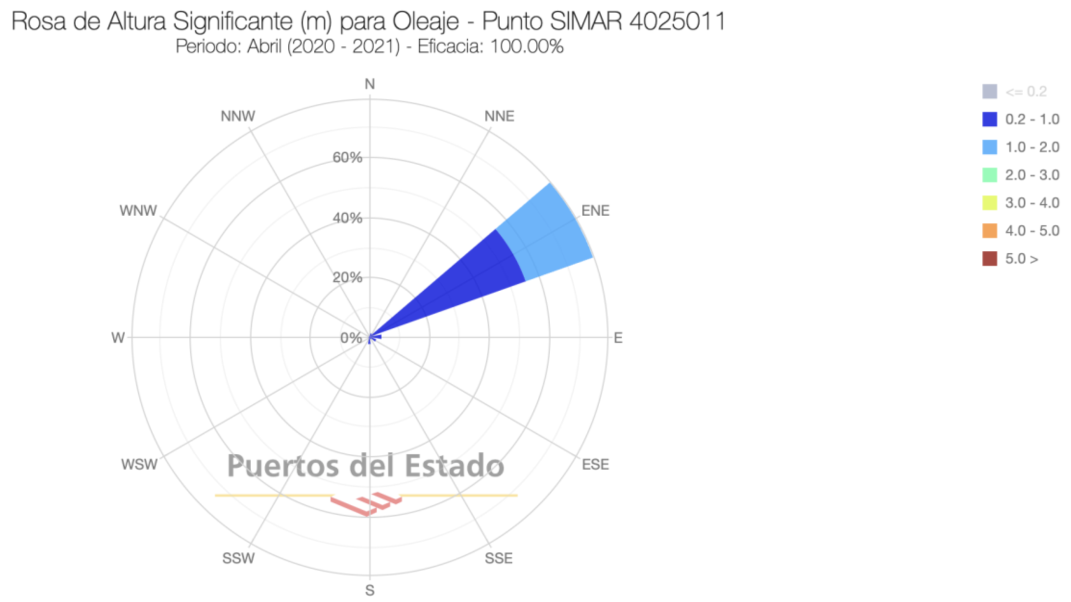

Figure S4: Rose of significant wave height regarding Playa Grande beach form April 2020 since April 2021.

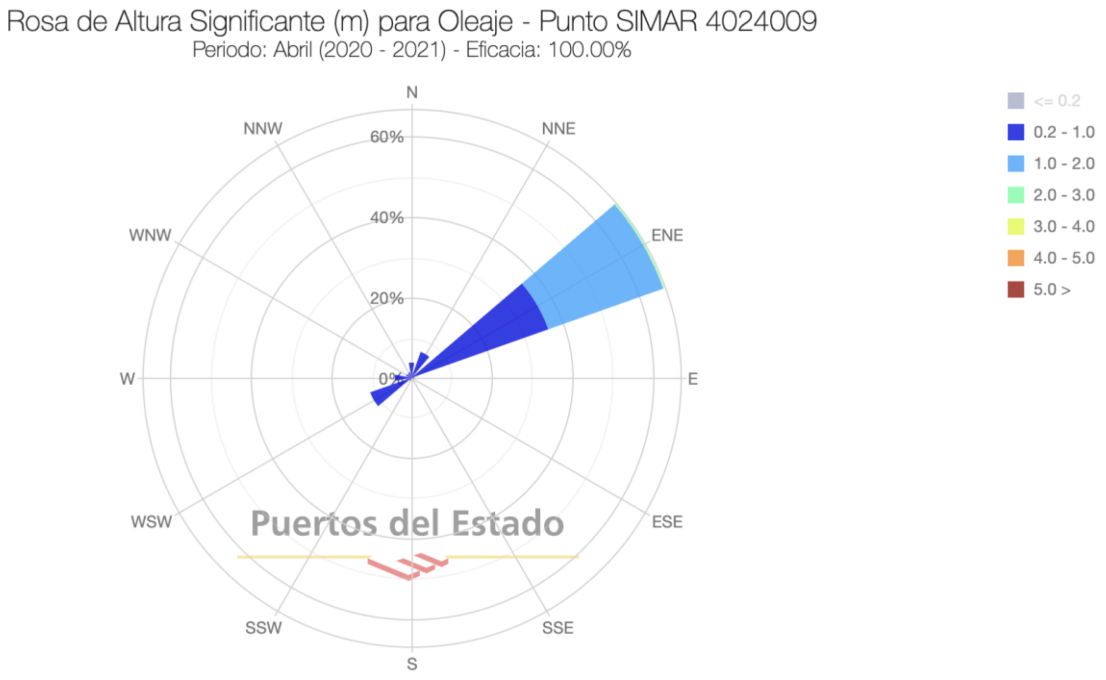

Figure S5: Rose of significant wave height regarding Punt del Bocinegro beach form April 2020 since April 2021.

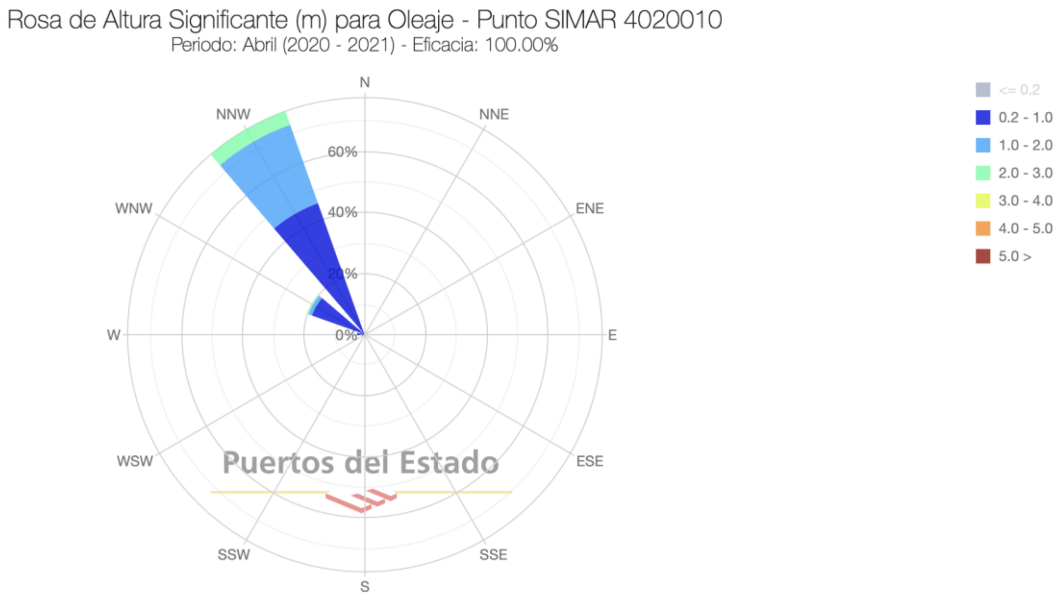

Figure S6: Rose of significant wave height regarding Puertito de Adeje beach form April 2020 since April 2021.

Supplementary Material File S3: Description of the wastewater discharges nearest to the beaches sampled (Compiled by authors based on data from GRAFCAN, 2022).

Beach	**Waste water ID number**	**Treatment**	**Wastewater effluent**	**Wastewater origin**	**Linear length beach-wastewater**
Almáciga	16TFSC	No information available (not authorized waste)	Continuous	Waste treatment plant into a well (56 m^3/h)	2200 m
Teresitas	23TFSC	Screening	Discontinuous. Emergency effluent waste water treatment plant	Waste water sewage spillway	500 m
	02TFSC	Screening	Discontinuous. Emergency effluent sewage pumping	Waste water sewage pumping spillway	523 m
La Viuda	07TFCA	Screening	Discontinuous. Emergency effluent sewage pumping	Waste water sewage pumping spillway	42 m
El Socorro	04TFAF	Secondary treatment	Continuous (574 m^3/h)	Waste water treatment plant	1063 m
	01TFAF	Secondary treatment	Continuous (576 m^3/h)	Waste water treatment plant	1032 m
	03TFAF	Screening	Discontinuous.	Industrial and urban waste water treatment plant	858 m

	02TFAT	Screening	Discontinuous.	Industrial and urban waste water sewage pumping spillway	434 m
Punta del Bocinegro	03TFGR	No information available (not authorized waste)	Discontinuous.	Waste water sewage pumping spillway	1130 m
	09TFGR	Screening	Discontinuous.	Waste water sewage pumping spillway	670 m
Puertito de Adeje	08TFAD	No information available (authorization expired)	Discontinuous.	Waste water sewage pumping spillway	1680 m
	09TFAD	No information available (authorization expired)	Discontinuous.	Waste water sewage pumping spillway	1830 m
	12TFAD	No information available (authorization expired)	Discontinuous.	Waste water sewage pumping spillway	1980 m

Figures of the wastewater discharges nearest to the beaches sampled (GRAFCAN, 2022)

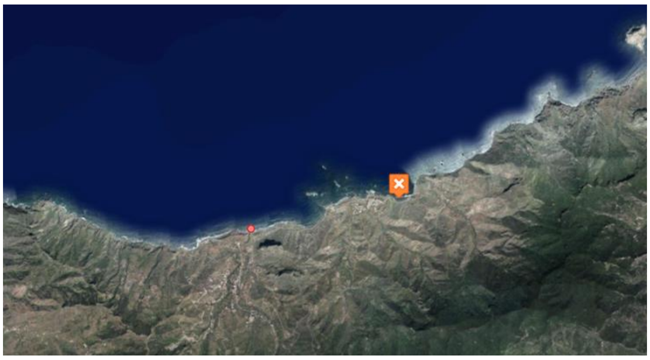

Figure S1: Almáciga beach sampling point (white cross on orange background) and nearby wastewater discharge (red dot).

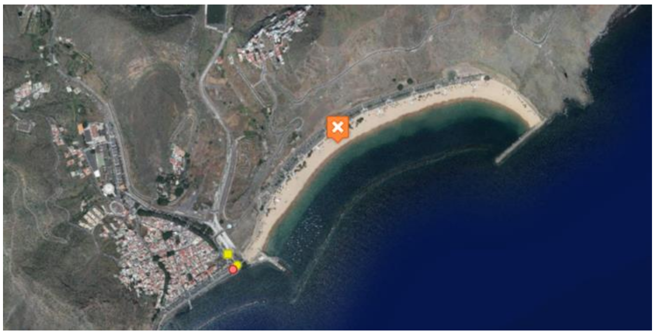

Figure S2: Teresitas beach sampling point (white cross on orange background) and nearby discontinuous wastewater discharge (red and yellow dots).

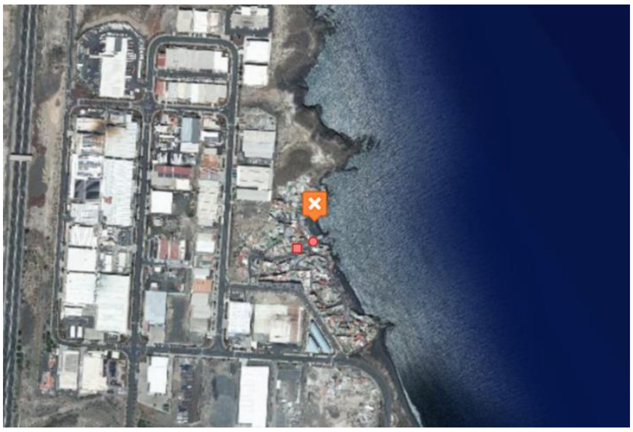

Figure S3: La Viuda beach sampling point (white cross on orange background) and nearby wastewater discharge (red dot).

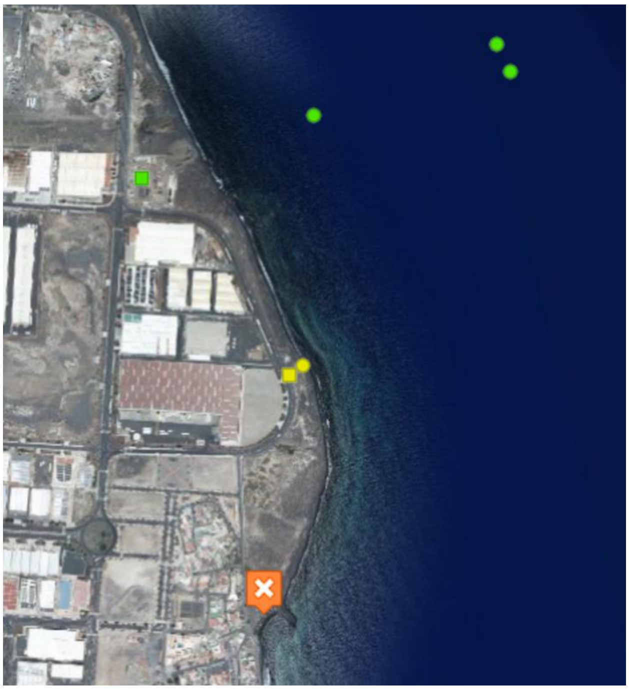

Figure S4: El Socorro beach sampling point (white cross on orange background) and nearby wastewater discharge (yellow and green dots).

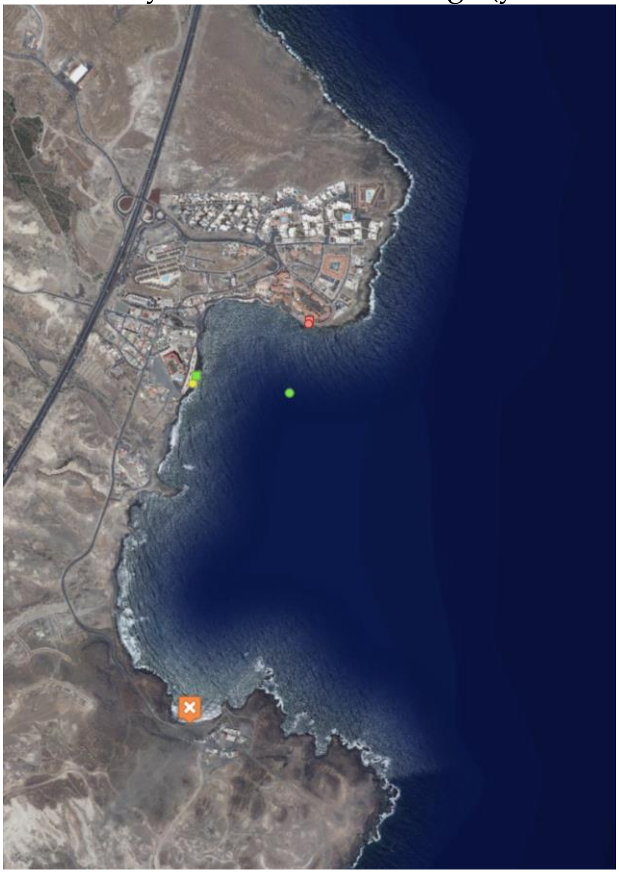

Figure S5: Playa Grande beach sampling point (white cross on orange background) and nearby wastewater discharge (yellow, green and red dots).

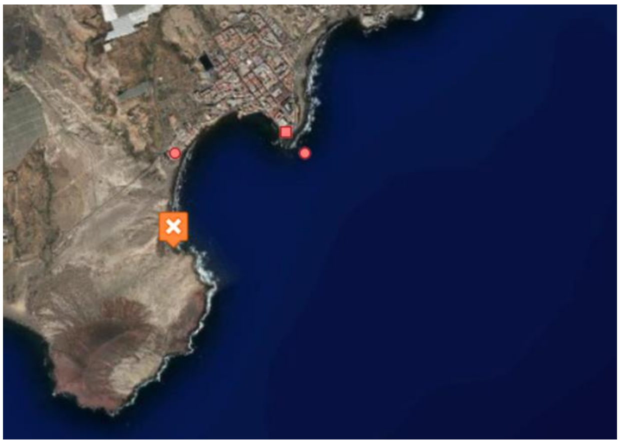

Figure S6: Punta del Bocinegro beach sampling point (white cross on orange background) and nearby wastewater discharge (red dots).

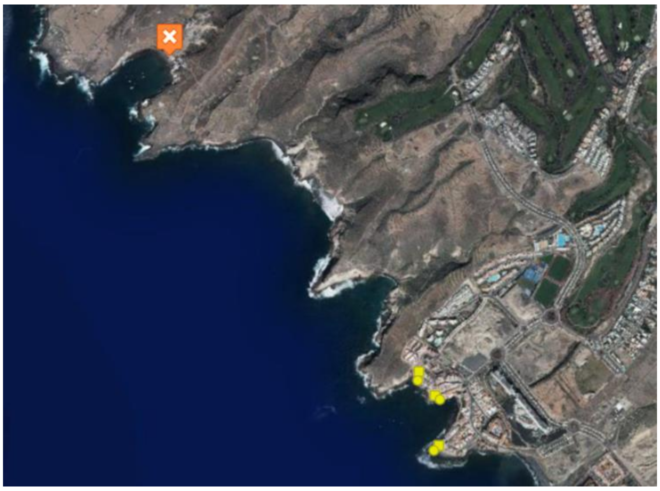

Figure S7: El Puertito beach sampling point (white cross on orange background) and nearby wastewater discharge (yellow dots).
